# Inferior Cerebellar Hypoplasia Resembling a Dandy-Walker-Like Malformation in Purebred Eurasier Dogs with Familial Non-Progressive Ataxia: A Retrospective and Prospective Clinical Cohort Study

**DOI:** 10.1371/journal.pone.0117670

**Published:** 2015-02-10

**Authors:** Filipa Bernardino, Kai Rentmeister, Martin J. Schmidt, Andreas Bruehschwein, Kaspar Matiasek, Lara A. Matiasek, Alexander Lauda, Heinz A. Schoon, Andrea Fischer

**Affiliations:** 1 Centre for Clinical Veterinary Medicine, Clinic of Small Animal Medicine, Ludwig Maximilian University, Munich, Germany; 2 Tierärztliche Praxis für Neurologie, Dettelbach, Germany; 3 Department of Veterinary Clinical Science, Small Animal Clinic, Justus-Liebig-University, Giessen, Germany; 4 Centre for Clinical Veterinary Medicine, Clinic of Small Animal Surgery and Reproduction, Ludwig Maximilian University, Munich, Germany; 5 Centre for Clinical Veterinary Medicine, Section of Clinical and Comparative Neuropathology, Ludwig Maximilian University, Munich, Germany; 6 Institute of Pathology, Faculty of Veterinary Medicine, University of Leipzig, Leipzig, Germany; Hospital Nacional de Parapléjicos—SESCAM, SPAIN

## Abstract

Cerebellar malformations can be inherited or caused by insults during cerebellar development. To date, only sporadic cases of cerebellar malformations have been reported in dogs, and the genetic background has remained obscure. Therefore, this study`s objective was to describe the clinical characteristics, imaging features and pedigree data of a familial cerebellar hypoplasia in purebred Eurasier dogs. A uniform cerebellar malformation characterized by consistent absence of the caudal portions of the cerebellar vermis and, to a lesser degree, the caudal portions of the cerebellar hemispheres in association with large retrocerebellar fluid accumulations was recognized in 14 closely related Eurasier dogs. Hydrocephalus was an additional feature in some dogs. All dogs displayed non-progressive ataxia, which had already been noted when the dogs were 5 – 6 weeks old. The severity of the ataxia varied between dogs, from mild truncal sway, subtle dysmetric gait, dysequilibrium and pelvic limb ataxia to severe cerebellar ataxia in puppies and episodic falling or rolling. Follow-up examinations in adult dogs showed improvement of the cerebellar ataxia and a still absent menace response. Epileptic seizures occurred in some dogs. The association of partial vermis agenesis with an enlarged fourth ventricle and an enlarged caudal (posterior) fossa resembled a Dandy-Walker-like malformation in some dogs. Pedigree analyses were consistent with autosomal recessive inheritance.

## Introduction

Cerebellar malformations can be inherited or acquired from insults during cerebellar development. Evidence for inherited cerebellar hypoplasia in dogs is rare in the veterinary literature and limited to few observations in wire-haired Foxterriers, Irish setters and Chow-Chows [1–3]. The most frequently documented etiologic factor in cerebellar malformations in the veterinary literature is viral infection of the developing cerebellum [4,5], but attempts to amplify parvovirus have failed in dogs with midline malformations and vermian defects resembling a Dandy-Walker malformation (DWM) [4].

A classic Dandy-Walker malformation (DWM) consists of complete or partial agenesis of the cerebellar vermis with an upward displacement and rotation of the remnants of the vermis, cyst-like dilation of the fourth ventricle and enlargement of the posterior fossa, with upward displacement of the tentorium cerebelli osseum, transverse sinuses, and torcula. Hydrocephalus may occur in up to 80% [6–10]. In many cases, no substantial enlargement of the posterior fossa is observed, which has led to the introduction of the term “Dandy-Walker variant” [10]; however, no consensus on this terminology exists in the literature [9,11,12], and the use of this term has been discouraged [11,13–15]. Recent evidence suggests that DWM and related malformations may represent a continuum and a classification system based on embryonic development and genotype has been proposed [16,17]. With this classification system, DWM and related malformations are classified as disorders of mesenchymal-neuropithelial signaling whereas VLDLR and reelin pathway mutations are classified as malformations of neuronal migration that predominantly affect the cerebellum and brainstem [16].

Anecdotal reports have described sporadic cases of vermis hypoplasia/agenesis resembling DWM with or without associated focal or generalized hypoplasia of the cerebellar hemispheres in dogs [18–23]. Reported affected breeds include the Miniature Schnauzer [1,19], Golden Retriever [21], Boston Terrier [18,23], Cocker Spaniel [20], Labrador Retriever, Bull Terrier, Weimaraner, Dachshund, Mixed Breed [22], Beagle, Silky Terrier [24], Wire-haired Miniature Dachshund [25], Chow-Chow and Tervueren [26]. The presence of a midline malformation with some resemblance to DWM in humans has been frequently suggested. To date, evidence for inheritance has been poor and limited to observations in Boston Terriers [18,23].

Therefore, the aim of the present investigation was to describe the clinical phenotype and imaging findings of familial non-progressive ataxia and cerebellar hypoplasia resembling a Dandy-Walker-like malformation (DWLM) in purebred Eurasier dogs. We provide a comprehensive clinical picture of this cerebellar malformation, which includes assessments of the clinical course and lifespan of the dogs, and propose autosomal recessive inheritance as. A VLDLR gene mutation was recently identified based on the data provided in this investigation [27].

## Materials and Methods

Retrospective and prospective case cohort studies were performed in Eurasier dogs with neurological signs.

Retrospective case review: The local Eurasier club (K.Z.G. Eurasier) provided records from 23 dogs with neurological symptoms. In individual cases, the local veterinarian was contacted for additional information.

Prospective investigations: The owners of nine purebred Eurasier dogs with neurological signs of ataxia and/or epileptic seizures were contacted by the Eurasier club and were encouraged to pursue neurological examination and advanced brain imaging by dedicated specialists. Each dog was subjected to clinical and neurological examinations by a board-certified veterinarian, and each dog underwent advanced neuroimaging. Magnetic resonance imaging (MRI) of the head (eight dogs) was performed on the anesthetized dogs using a 1.5 Tesla scanner (Magnetom Symphony Syngo MR, Siemens AG, Erlangen, Germany; four dogs) or a 1.0 Tesla scanner (Gyroscan Intera, Philips, Hamburg, Germany; four dogs). T1-weighted (spin echo repetition time [TR], 482 ms; minimum echo time [TE], 15 ms; signal averaging [NSA], 4; slice thickness, 4 mm; interslice gap, 0.4 mm) and T2-weighted turbo-spin echo (TR, 4146 ms; TE, 108 ms; slice thickness, 2 mm; 448 × 448 matrix; FOV, 130 × 130) images were acquired in the sagittal, dorsal, and transverse planes, and fluid attenuation inversion recovery (FLAIR) and T2 gradient echo (GE) sequences were obtained in the transverse plane. A gadolinium-based contrast agent (Omniscan, 0.1 mmol/kg) was administered intravenously. In one animal, computed tomography (CT) of the head was obtained by multislice CT (Somatom Balance, Siemens, Erlangen, Germany) (120 kV; 350 mA; matrix, 512 × 512; slice thickness, 2 mm; pitch, 1). MRI was also performed on three littermates and the dam and sire of one affected dog (dog 6; litter 4), the dam of another affected dog (dog 9, litter 6), and CT was performed on the dam of two affected littermates (dogs 7, 8; litter 5) to ensure the dogs` health status prior to breeding.

All MRI and CT brain images and histological specimens were reviewed. The presence or absence of the midline cerebellar vermis structures was assessed. Special care was taken to assess the position of the tentorium cerebelli. The size of the caudal fossa was assessed visually in each dog by independent examinations of a board-certified radiologist and two board-certified neurologists and, thereafter, morphometrically as the ratio of the caudal fossa area to the total braincase area on midsagittal T2W brain images, as published previously (OsiriX; v.5.6 Pixmeo Sarl) [28].

The pedigrees were analyzed using electronic data files provided by the Eurasier club (Dog manager; Breeder Soft, Delmenhorst, Germany). The pedigrees were compiled to the first common ancestor in all dogs, using a human genealogy software program (Geno Pro 2011).

The clinical course and the life span of the dogs were assessed by information provided by the breed club and contact to the dogs` owners. Repeated neurological examinations and video documentations of the gait were performed on three dogs of the prospective cohort.

### Ethics Statement

All investigations were conducted in strict compliance with the restrictions of the German Animal Protection Law. The authors declare that prior approval was obtained from the respective breed club (Kynologische Zuchtgemeinschaft Eurasier e.V.) and the Clinic of Small Animal Medicine institutional review board. All dogs (purebred Eurasier dogs) lived with their owners and the owners of the dogs gave permission for their animals to be used in this study.

## Results

Inferior cerebellar hypoplasia resembling a DWLM was confirmed in 14 dogs. The main finding was absence of the caudal portions of the cerebellar vermis and the caudal aspects of the cerebellar hemispheres associated with an enlarged fourth ventricle in all dogs. An enlarged caudal (posterior) fossa consistent with a classic DWM was evident in three dogs (21%), and four dogs exhibited hydrocephalus (29%). The assessment was based on neuroimaging and morphometric measurements in eleven dogs (dogs 1–11) and on post mortem examinations in three dogs. PCR amplification of canine parvovirus type 2 nucleic acid was negative, and corpus callosum agenesis was an additional finding in these three dogs (dogs 12–14).

Other findings: Cerebellar hypoplasia with or without hydrocephalus was diagnosed during post-mortem examinations in three other dogs, but histological slides were not provided to us for secondary review. Other diagnoses were confirmed in six dogs: caudal fossa arachnoid cyst (one dog; MRI), hydrocephalus restricted to the supratentorial region (two dogs; MRI, histopathology), and idiopathic epilepsy (three dogs; MRI). No specific diagnoses were obtained for nine dogs with ataxia which were reported to the breed club before the dogs were eight weeks old.

### Signalment and neurological examination

Signalment and clinical data from 14 Eurasier dogs (6 male and 8 female dogs; 5 weeks to 4 years old at clinical presentation) with a confirmed diagnosis of inferior cerebellar hypoplasia resembling DWLM. are presented in Table 1. Non-progressive ataxia, which was noted at an early age when the dogs began to walk, was the predominant clinical sign in these 14 dogs. The frequencies of the main neurological features in each patient cohort are presented in Table 2.

**Table 1:** Clinical findings in Eurasier dogs with inferior cerebellar hypoplasia resembling a Dandy-Walker-like malformation (DWLM).

**Dog**	**Sex**	**Age at presentation**	**Presenting complaint**	**Neurological examination (abnormal findings)**	**Seizures**	**Clinical course and lifespan**
1	F	8 w	Ataxia, circling	Circling and moderate ataxia, rolling, falling	Yes (onset 1 y)	Non-progressive, 11 y (alive)
2	M	6 w	Ataxia, circling, and head tremors	Generalized ataxia, head tremors, reduced postural reactions (all limbs)	N/A	Improved, 6 y (alive)
3	F	5 w	Ataxia, head tremors	Generalized ataxia, hypermetric limb movements, head tremors	Yes (onset 4 y)	Improved ataxia, 6 y (alive)
4	F	5 w	Ataxia	Generalized ataxia, hypermetric limb movements, head tremors, reduced postural reactions (all limbs)	Yes (onset < 1 y)	Euthanasia at 10 m of age (due to poorly controlled seizures)
5	F	6 w	Pelvic limb weakness	Severe generalized ataxia, falling to the left, reduced postural reactions (proprioceptive positioning) all limbs, cervical pain	N/A	Euthanasia at 5 m of age (for unknown reasons)
6	F	6 w	Subtle incoordination, smaller size than littermates	Very mild dysmetric limb movements, truncal sway, subtle delay in initiation of postural reactions at initial presentation (unremarkable on follow-up examinations), oculocephalic movements with a subtle delay in medial eye movements, absent menace response on follow-up examination at 1 year of age	No	Non-progressive, 5 y (alive)
7	F	8 w	Ataxia and head tremors	Moderate generalized cerebellar ataxia of trunk and limbs with hypermetric limb movements, episodic falling, head tremors, horizontal nystagmus, absent menace response beyond 12 weeks of age	No	Improved ataxia, mild dysmetria. 2 y 10 m (alive)
8	M	8 w	Ataxia and head tremors	Moderate generalized cerebellar ataxia of trunk and limbs with hypermetric limb movements and episodic falling, head tremors, horizontal nystagmus, absent menace response beyond 12 weeks of age	No	Improved ataxia, mild dysmetria, 2 y 10 m (alive)
9	F	8 w	Ataxia, episodic rolling, problems calculating distances	Moderate cerebellar ataxia, rolling to the left and right side, hypermetric limb movements, head tremors (intention tremors), hypermetric hopping reactions (thoracic limbs), absent menace response beyond 12 weeks of age	No	Improved ataxia, 3 y 10 m (alive)
10	M	4 y 9 m	Recent onset of generalized seizures, non-progressive pelvic limb ataxia since 6 w aged	Mild pelvic limb ataxia, worse following exercise, mild ataxia of thoracic limbs when going downstairs, reduced menace response, horizontal jerk nystagmus and nystagmus with alternating directions, subtle delay in initiation of postural reactions, worse in the pelvic limbs	Yes (onset < 4 y)	Non-progressive, 6 y (alive)
11	F	9 w	Ataxia and inability to climb stairs	Mild generalized ataxia, mildly dysmetric gait, mild head tilt to the left, leaning to the left, subtle positional nystagmus	No	Improved, 8 y (alive)
12	M	5 w	Ataxia	Cerebellar ataxia	No	Euthanasia at 5 w of age
13	M	5 w	Ataxia	Ataxia characterized by mild dysmetria, worse in the pelvic limbs, and truncal sway	No	Euthanasia at 5 w of age
14	M	5 w	Ataxia	Ataxia characterized by mild dysmetria, worse in the pelvic limbs, and truncal sway	No	Euthanasia at 5 w of age

M. male; F: femaley: year; m: month; w: weeks; N/A: not assessed

**Table 2:** Frequencies of the main clinical features in the two patient cohorts.

**Clinical and neurological findings**	**Retrospective cohort (n = 8)**	**Prospective cohort (n = 6)**
Smaller size for breed standards	-	17.0%
Mild to moderate generalized ataxia	87.5%	100.0%
Severe generalized ataxia	12.5%	-
Dysmetric/hypermetric gait	50.0%	83.0%
Truncal sway	25.0%	17.0%
Circling	25.0%	-
Episodic falling and/or rolling	25.0%	50.0%
Leaning	-	17.0%
Head tilt	-	17.0%
Head tremors	37.5%	50.0%
Absent menace reaction >12 weeks of age	N/A	67.0%
Nystagmus	N/A	67.0%
Slow medial eye movements	N/A	17.0%
Proprioceptive deficits	37.5%	33.0%
Delayed initiation of hopping and wheelbarrowing reaction	N/A	50.0%
Hypermetric wheelbarrowing	N/A	33.0%
Seizures	37.5%	17.0%

Retrospective cohort (dogs 1–5, dogs 12–14, Table 1): All dogs presented with generalized ataxia. This ataxia was characterized by episodic falling and/or rolling or a hypermetric gait. Cerebellar ataxia was evident on provided video recordings (S1 Video). Additional signs described in some dogs were occasional head tremors, which resembled intention tremors and proprioceptive deficits. Three of these dogs developed epileptic seizures at one year of age (dog 1), four years of age (dog 3), or within the first year (dog 4).

Prospective cohort (dogs 6–11, Table 1): All dogs presented with non-progressive ataxia. Most notably, in one dog, ataxia was hardly visible on initial presentation and was detected as only truncal sway and subtle dysmetria to an experienced observer (dog 6, S2 Video). In the other dogs, the ataxia was specifically characterized as cerebellar ataxia with a hypermetric gait and symmetrical ataxia of the trunk and limbs (dogs 7 and 8, S3 Video), which improved in the adult dogs (S4 Video); cerebellar ataxia with episodic rolling to both sides (dog 9); a subtle dysmetric gait and pelvic limb ataxia (dog 10, S5 Video); and a subtle dysmetric gait of all limbs, mild head tilt and leaning to one side (dog 11, S6 Video). Other signs noted during the neurological examination were delayed initiation of the hopping and wheelbarrowing reactions (three dogs, S7 Video); hypermetric wheelbarrowing (two dogs); delayed postural reactions in a puppy, which were no longer evident in adulthood (one dog); absent menace reaction (six dogs); slow medial eye movements on examination of oculocephalic movements (one dog); and nystagmus (four dogs). The last was described as positional nystagmus (one dog), alternating horizontal jerk nystagmus and erratic nystagmus (one dog, S8 Video) or horizontal nystagmus (two dogs). The menace reaction remained absent or reduced in four dogs beyond 12 weeks. One dog was presented for generalized epileptic seizures at four years of age (dog 10).

### Imaging

Retrospective cohort (dogs 1–5, Table 3): In all dogs, a uniform cerebellar malformation characterized by the absence of caudal portions of the cerebellar vermis and symmetrical hypoplasia of the cerebellar hemispheres was identified. Large retrocerebellar fluid accumulations associated with an enlarged fourth ventricle were identified on midsagittal views (dog 4, S1 Fig.). A butterfly shape of the cerebellum corresponding to remnants of the cranial aspects of the vermis and cerebellar hemispheres was evident on dorsal views. The reduced size of the cerebellar vermis was more accurately assessed on MR than on CT images. Cranial displacement of the tentorium cerebelli and an enlarged caudal fossa were evident in two dogs (dogs 3 and 5) of the retrospective cohort. Additional supratentorial anomalies included hydrocephalus internus (3 dogs), asymmetric lateral ventricles (1 dog), and a poorly visible corpus callosum (1 dog).

**Table 3:** Neuroimaging findings in Eurasier dogs with familial non-progressive ataxia and inferior cerebellar hypoplasia resembling a DWLM.

**Dog**	**Caudal fossa imaging findings**	**Hydrocephalus**	**Size of caudal fossa**
1	MRI—Absent caudal portion of the cerebellar vermis, enlarged size of the fourth ventricle, retrocerebellar fluid accumulations, absent caudal parts of the cerebellar hemispheres, normal positioned tentorium cerebelli osseum	None	Unremarkable
2	MRI—Large caudal fossa fluid accumulation dorsal to the brainstem, no visible cerebellar structure	Severe hydrocephalus internus (lateral ventricles), septum pellucidum not visible	Unremarkable
3	CT—Large hypodensity suggestive of fluid accumulation in the caudal fossa, visible tissue remnants rostrally in the caudal fossa. these present in a butterfly shape with rostrodorsal and ventral fluid accumulations dorsal to the brainstem on transverse sections, elevated tentorium cerebelli osseum (	Moderate hydrocephalus internus (lateral ventricles)	Increased
4	MRI—Absent caudal portions of the cerebellar vermis, absent caudal parts of the cerebellar hemispheres, large cyst-like appearance of the fourth ventricle, cyst extends into the retrocerebellar region, caudal border outlined by a linear structure with soft tissue density on midsagittal T2-weighted images	Moderate unilateral hydrocephalus with asymmetric lateral ventricles, corpus callosum poorly identifiable	Unremarkable
5	CT—Large hypodensity in the caudal fossa suggestive of fluid accumulation, visible tissue remnants rostrally and dorsally in the rostral third of the caudal fossa, these present in a butterfly shape with rostrodorsal and ventral fluid accumulations dorsal to the brainstem in the midline on transverse sections, elevated tentorium cerebelli osseum^*^	Moderate hydrocephalus internus (lateral ventricles, third ventricle), small quadrigeminal cyst	Increased
6	MRI—Absent caudal portions of the cerebellar vermis and cerebellar hemispheres with associated large retrocerebellar fluid accumulations, rostrodorsal portions of the cerebellar hemispheres preserved, fourth ventricle appears enlarged, caudally in the caudal fossa a thin band-like structure traversing the caudal fossa in a ventrodorsal direction; other findings: subtentorial flattening and thickening of the supraoccipital bone resulting in a triangular shape*	None, septum pellucidum not visible	Unremarkable
7	MRI—Absent caudal portions of the cerebellar vermis and cerebellar hemispheres, large retrocerebellar fluid accumulations, rostrodorsal portions of the cerebellar hemispheres preserved, fourth ventricle appears enlarged on midsagittal views, thin band-like structure in the caudal fossa at the level of the most caudal extend of the cerebellar hemisphere remnants; other findings: subtentorial flattening and thickening of the supraoccipital bone	None, subjectively thinned appearance of corpus callosum	Unremarkable
8	MRI—Absent caudal portions of the cerebellar vermis and cerebellar hemispheres, with associated large retrocerebellar fluid accumulations, rostrodorsal portions of the cerebellar hemispheres preserved, fourth ventricle appears enlarged on midsagittal views with thin band-like structure in the caudoventral caudal fossa; other findings: thin band-like structure in the caudoventral caudal fossa, subtentorial flattening and thickening of the supraoccipital bone	None, subjectively thinned appearance of corpus callosum	Unremarkable
9	MRI—Absent caudal portions of the cerebellar vermis and cerebellar hemispheres with associated large retrocerebellar fluid accumulations, rostrodorsal portions of the cerebellar hemispheres preserved with indiscernible foliae, sulci and fissures and ill-defined grey-white matter transition. Fourth ventricle appears enlarged on midsagittal views and continuous with retrocerebellar fluid accumulations on dorsal views. Other findings: thin lamellar structure in the caudoventral caudal fossa; subtentorial flattening and thickening of the supraoccipital bone resulting in a triangular shape and an irregular caudodorsal contour of the caudal fossa	None, subjectively thinned appearance of corpus callosum	Unremarkable
10	MRI—Absent caudal portions of the cerebellar vermis and the cerebellar hemispheres with associated large retrocerebellar fluid accumulations, rostrodorsal portions of the cerebellar hemispheres preserved with indiscernible foliae, sulci and fissures and ill-defined grey-white matter transition. Fourth ventricle appears enlarged on midsagittal views, but continuous with retrocerebellar fluid accumulations on dorsal views. Other findings: thin lamellar structure in the caudoventral caudal fossa; subtentorial flattening and thickening of the supraoccipital bone resulting in a triangular shape and an irregular caudodorsal contour of the caudal fossa	Mild hydrocephalus internus (lateral ventricles), septum pellucidum not visible	Unremarkable
11	CT—Large hypodensity in the caudal fossa suggestive of fluid accumulation, visible tissue remnants rostrally in the caudal fossa. Tissue remnants present in a butterfly shape with rostrodorsal and ventral fluid accumulations in the midline on transverse sections; enlargement of the caudal fossa and elevated tentorium cerebelli, bilateral symmetric osseus lamina protruding into the caudal fossa from rostrodorsal creating the impression of a split tentorium	Severe hydrocephalus (lateral ventricles), quadrigeminal cyst	Increased

MRI: magnetic resonance images; CT: computed tomographic images

**Fig 1:**
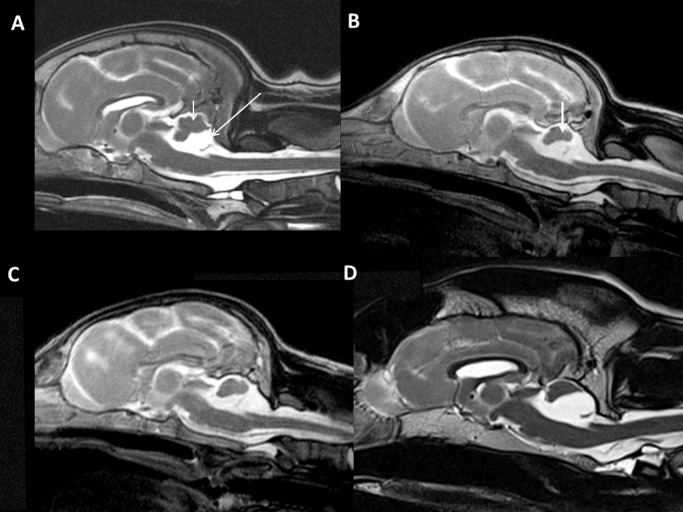
Midsagittal MR brain images. Midsagittal MR brain images (T2-weighted) of the affected Eurasier dogs. In all dogs, a uniform cerebellar malformation was identified, characterized by absence of the caudal portions of the cerebellar vermis and, to a lesser degree, the caudal aspects of the cerebellar hemispheres and large caudal (posterior) fossa fluid accumulations (S2 –S6 Figs.). The fourth ventricle appeared enlarged on midsagittal views and continuous with large retrocerebellar fluid accumulations (large arrow). Tissue remnants in the rostrodorsal caudal fossa correspond to the rostral portions of the cerebellar vermis (small arrow). Note the cyst-like appearance of the fourth ventricle in D. A: dog 6; B: dog 7; C: dog 9; D: dog 10.

**Fig 2:**
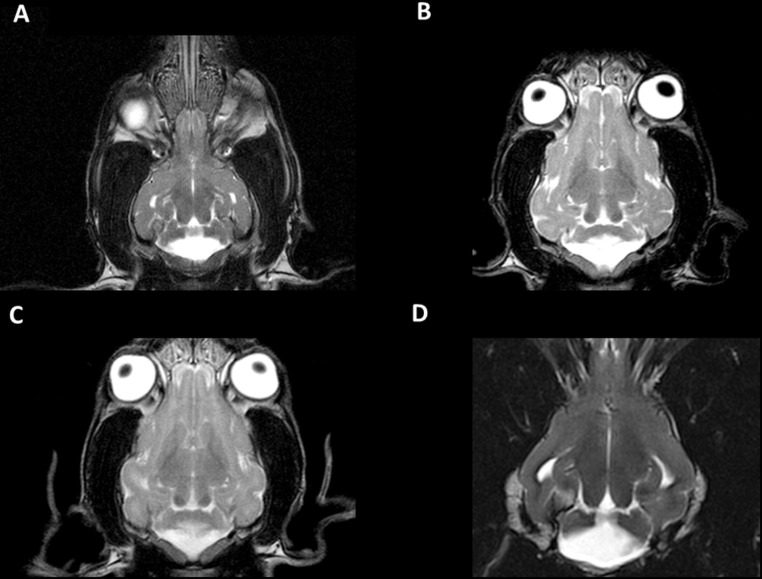
Dorsal MR brain images. Dorsal MR brain images (T2-weighted) of the affected Eurasier dogs reveal a prominent midline defect with absent caudal portions of the cerebellar vermis (midline) and cerebellar hemispheres (lateral) in association with a large retrocerebellar fluid accumulation. A: dog 6; B: dog 7; C: dog 9; D: dog 10. Images A and D are located more ventrally than B and C.

**Fig 3:**
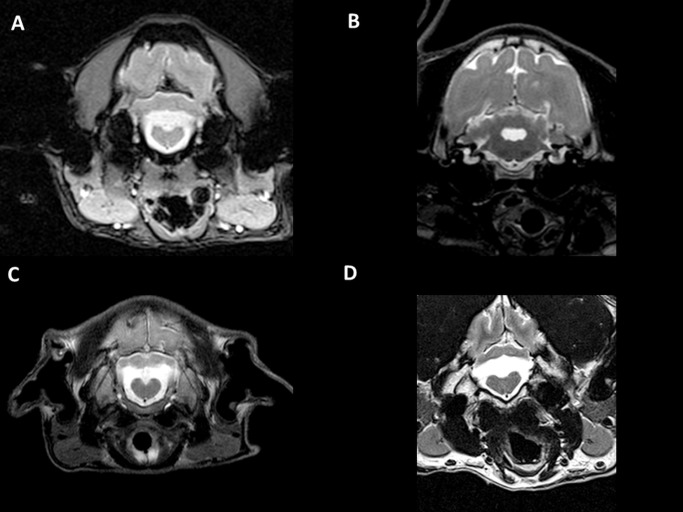
Transverse MR brain images. Transverse MR brain images (T2-weighted) of four affected Eurasier dogs at the level of the cerebellar peduncles (B) and medulla oblongata (A, C, D). The myelencephalon appears unremarkable. The fourth ventricle has a cyst-like appearance in the rostral sections (B) and is continuous with retrocerebellar cerebrospinal fluid accumulations in the more caudal sections (A, C, D). A: dog 6; B: dog 7; C: dog 9; D: dog 10.

**Fig 4:**
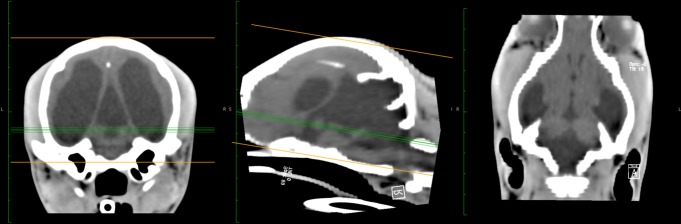
CT brain images. Computed tomographic images of dog 11, featuring transverse views of the brain at the level of the tympanic bullae, midsagittal and dorsal views. Images show hydrocephalus of the lateral ventricles, supracollicular fluid accumulation (“quadrigeminal cyst”) and large fluid accumulations in the caudal fossa dorsal to the brainstem. The cerebellar remnant tissue in the caudal fossa has a butterfly shape consistent with the loss of midline cerebellar vermis structures. The caudal fossa appears enlarged.

Prospective cohort (dogs 6–11, Table 3, Figs. 1–4): In all dogs, a uniform cerebellar malformation was identified that was characterized by the absence of the caudal portions of the cerebellar vermis and, to a lesser degree, the caudal aspects of the cerebellar hemispheres as well as large caudal (posterior) fossa fluid accumulations (S2–S6 Figs.). The fourth ventricle appeared enlarged on midsagittal views and contiguous with large retrocerebellar fluid accumulations (Fig. 1, Fig. 2; Fig. 3; S2–S6 Figs.). A thin structure traversing the fluid-filled spaces in the caudal portion of the caudal fossa was frequently identified on midsagittal views (Fig. 1A-D) and less consistently on dorsal views (Fig. 2B). The tentorium cerebelli was displaced rostrodorsally, and the caudal fossa appeared enlarged in one dog (dog 11, Fig. 4). Additional supratentorial findings, such as hydrocephalus (1 dog) or ventriculomegaly (1 dog), a poorly visible or subjectively thinned corpus callosum (3 dogs), and an absent septum pellucidum (2 dogs), were identified. MRI scans of littermates and both parents of litter 4 (dog 6), the dam of litter 5 (dogs 7 and 8) and the dam of litter 6 (dog 9) failed to reveal any structural abnormalities.

**Fig 5:**
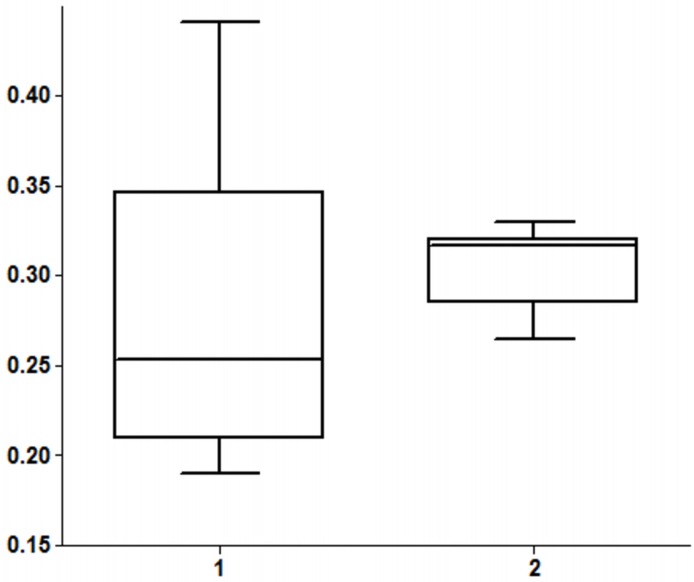
Midline caudal fossa ratio in Eurasier dogs with (1) and without (2) cerebellar hypoplasia. Boxplots demonstrate wide variations in midline caudal fossa ratio in Eurasier dogs with inferior cerebellar hypoplasia resembling DWLM (1: range 0.191–0.441; n = 9) compared to Eurasier dogs with unremarkable brain images (2: range 0.2645–0.3300; n = 10). Midline caudal fossa ratio was increased in three dogs with inferior cerebellar hypoplasia resembling DWLM (S1 Table).

Morphometric measurements (Fig. 5, S1 Table): Calculation of the ratio of the caudal fossa area to the total braincase area on midsagittal images indicated wide variation of the caudal fossa size (n = 10; range 0.191–0.441; mean ± SD 0,287±0.074) with enlargement of the caudal fossa in three dogs with cerebellar hypoplasia (dog 3: 0.441; dog no. 5: 0.344; dog no. 11: 0.354) compared with their unaffected littermates and parents (n = 9; range 0.265–0.330; mean ± SD 0,305±0.021) and with the published ratios in other dog breeds [28].

### Pedigree analysis

**Fig 6:**
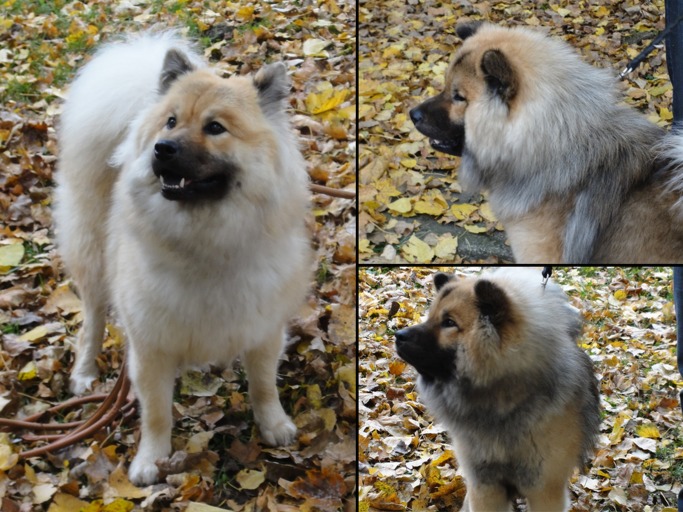
Eurasier dog breed.

**Fig 7:**
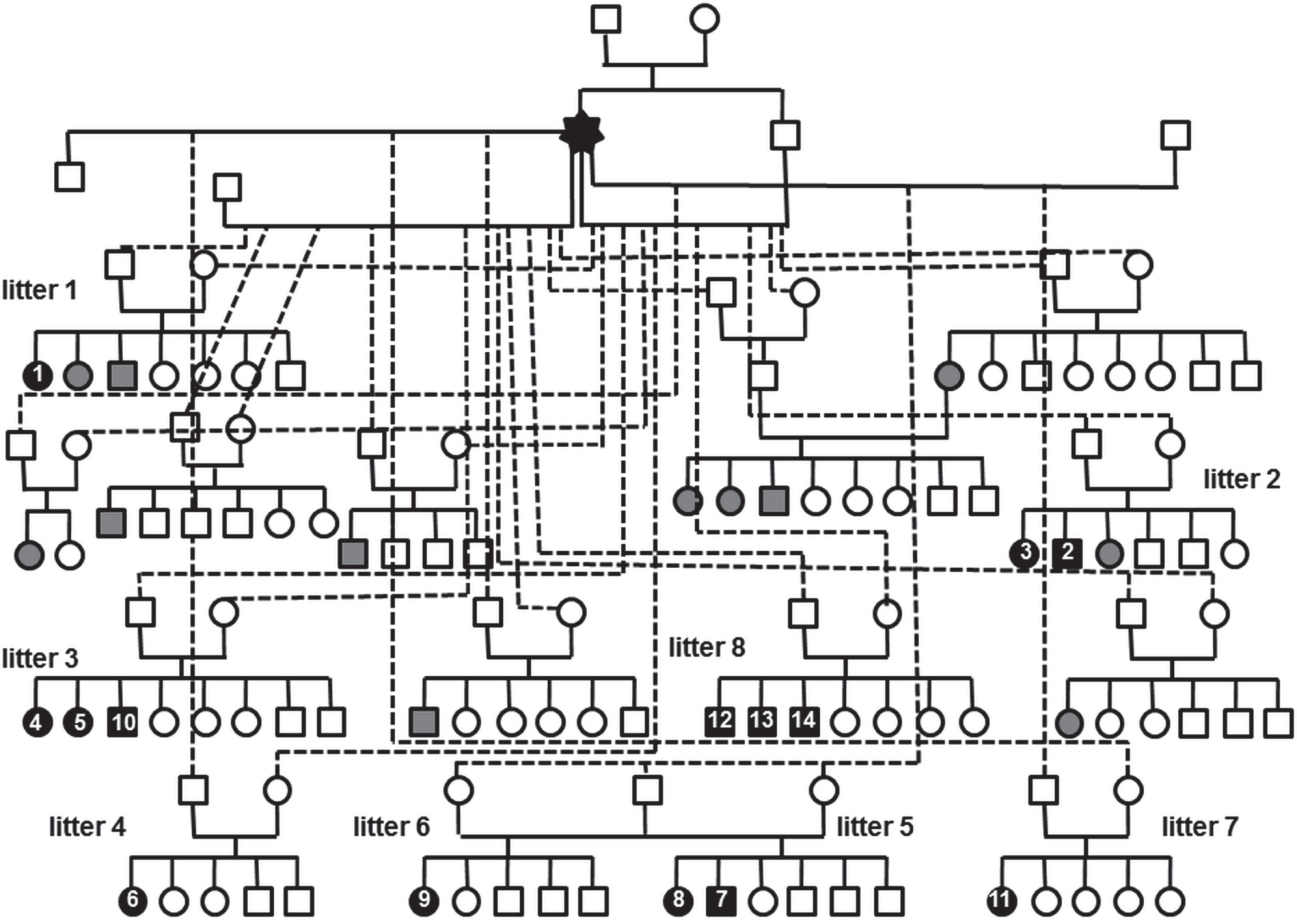
Pedigree. Pedigree of Eurasier dogs with familial non-progressive ataxia and cerebellar hypoplasia resembling a Dandy-Walker like malformation (DWLM). Female dog, ○; male dog, □; black, confirmed cases; the numbers refer to the dog numbers in Tables 1 and 2; gray, suspected cases, based on clinical signs, not confirmed by imaging. All cases could be traced to a common female founder.

Pedigree analysis suggested autosomal recessive inheritance. Eight litters with single or multiple affected Eurasier dogs (Fig. 6) of both sexes were reported (median 26.5%–35.5% affected) (Fig. 7). Litter 1: dog 1 belonged to a litter of seven, and non-progressive ataxia was reported in two other dogs from this litter (14%–43% affected). Litter 2: dogs 2 and 3 belonged to a litter of six, and non-progressive ataxia was reported in one additional littermate (33%–50% affected). Litter 3: dogs 4, 5, and 10 were from a litter of eight (38% affected). In this litter, the caudal fossa appeared enlarged in one affected dog (dog 5) and was unremarkable in the two other affected dogs from the same litter. Litter 4: dog 6 was the only confirmed case in a litter of five (20% affected). Litter 5: dogs 7 and 8 were from a litter of six (33% affected). Litter 6: dog 9 presented from a litter of five (20% affected). Litter 7: dog 11 was the only confirmed case in a litter of five (20% affected). Litter 8: dogs 12, 13 and 14 were from a litter of seven dogs (43% affected). All affected dogs (n = 14) were traced back to a common founder, a female dog introduced into the breeding program in 1972 (Fig. 7).

### Clinical course and life span

Five dogs were euthanized as puppies or within their first year of life. One of these was euthanized at 10 months because of severe seizures which were refractory to treatment with antiepileptic drugs. The other nine dogs were still alive at study conclusion (age 2.8 to 11 years; median 6 years). The ataxia was non-progressive in all nine dogs and improvement was reported in six dogs (Table 1). Video documentation was obtained from two dogs which had shown severe ataxia as puppies (S3 Video) and considerable improvement of the ataxia in adulthood (S4 Video). Four dogs developed epileptic seizures. No change in behavior, attitude or mental status was appreciated by the owners in any of the dogs, and all dogs were considered completely functional pets.

## Discussion

Cerebellar hypoplasia resembling DWLM was diagnosed in 14 closely related purebred Eurasier dogs with familial non-progressive ataxia. The similar imaging phenotype, the existence of multiple affected dogs in several litters, and the fact that all cases were traced to a common founder suggested an inherited developmental defect. A VLDLR receptor mutation was subsequently identified in these dogs [27].

Affected Eurasier dogs displayed non-progressive ataxia with an early onset when the dogs began to ambulate. Other clinical signs were nystagmus, an absence of the bilateral or unilateral menace reaction beyond 10–12 weeks of age, and epileptic seizures occurring later in life in some dogs. Thus the clinical phenotype of the dogs reflects VLDLR-associated cerebellar hypoplasia in humans, who display severe truncal ataxia (dysequilibrium syndrome) as the main clinical manifestation [29–35]. Similar to our dogs peripheral ataxia of the limbs, loss of smooth pursuit eye movements and epileptic seizures occur in some humans with VLDLR-associated cerebellar hypoplasia [29,33]. More clinical heterogeneity is recognized in human patients with DWM and its variants; these individuals may exhibit a variety of clinical features ranging from none to neurocognitive deficits, motor delay, hypotonia, speech delay, autistic features, ocular symptoms including nystagmus and strabismus to cerebellar ataxia, dizziness, and epileptic seizures [6,36–39] It is of interest that the dogs` carers did not recognize any relevant neurobehavioral signs or mental retardation in their dogs while neurocognitive deficits are frequently associated with VLDLR-associated cerebellar hypoplasia and also with DWM and its variants. Specifically, individuals with VLDLR-receptor associated cerebellar hypoplasia may never gain the ability to speak and remain mentally retarded [29–35]. While this is in contrast to the observations in our dogs, we cannot exclude that the dogs` carers did not appreciate mild to moderate mental retardation in their dogs or that mental retardation was missed in dogs of the retrospective study part.

In some dogs, the gait abnormalities persisted throughout life, while marked improvement was noted in others that had displayed severe cerebellar ataxia of the trunk and limbs as puppies (S3 Video) and showed only subtle gait abnormalities in adulthood (S4 Video). These findings support the presence of compensatory mechanisms or plasticity of the remaining cerebellum. The quadrupedal locomotion in some humans with VLDLR-associated cerebellar hypoplasia is explained by behavioral adaptation to the severe orthostatic instability [33,35]. As dogs walk on their four legs with a lower center of gravity, dogs may be able to adapt more easily. Repeated measurements of the degree of the ataxia should be highly valuable in future cases of cerebellar malformations in dogs and humans but were not assessed as part of this study. An absent menace reaction was considered an important indicator of cerebellar disease in affected Eurasier dogs older than twelve weeks with mild symptoms or barely visible ataxia. Thus the menace reaction should be carefully evaluated in any Eurasier dog that has a subtle gait disturbance and has not yet undergone genetic testing or MR screening for identification of a cerebellar malformation. Epileptic seizures developed later in life in some affected dogs. The seizures appeared unrelated to the presence of hydrocephalus and partial agenesis of the corpus callosum and we did not appreciate lissencephaly. Detailed assessment for other MR correlates of seizures was impaired due to lack of appropriate breed and age-matched MR controls. Epileptic seizures occur in a proportion of humans with VLDLR-associated cerebellar hypoplasia [29,33], and also with DWM and related malformations [6,36–39].

Abscence of the caudal portions of the cerebellar vermis and the caudal portions of the cerebellar hemispheres in association with large caudal fossa fluid accumulation were the main imaging finding in each Eurasier dog. There was close correlation between the specific malformation and the clinical signs because all affected dogs showed signs of non-progressive cerebellar disease, mainly ataxia and dysmetria. Additional vestibular signs like head tilt in one dog and nystagmus may be explained by flocculondular lobe involvement. The menace reaction may be abolished by diffuse cerebellar disorders [1,22]. Thus the cerebellar hypoplasia in our dogs mirrors closely the inferior cerebellar hypoplasia described in humans in association with VLDLR mutations [29–35]. The very low-density lipoprotein receptor is part of the reelin pathway which modulates neuronal migration in the cerebral cortex and cerebellum [1,40]. The different types of VLDLR mutations in humans share a unique pathologic picture characterized by inferior cerebellar hypoplasia with absence of the caudal (posterior) portions of the cerebellar vermis and the cerebellar hemispheres. Other pathological features are a small pons and a simplified cortical sulcation pattern in humans [33]. Yet additional pathologic features with resemblance to classic DWM were present in a proportion of dogs. We observed wide variation in caudal fossa size with an enlarged caudal fossa supportive of classic DWM in three dogs, a normal sized caudal fossa in others and both phenotypes within the same litter (Fig. 5, S1 Table). Enlargement of the posterior fossa is required for the diagnosis of classic DWM in humans and differentiates classic DWM from isolated vermis agenesis/hypoplasia and generalized hypoplasia of the cerebellar vermis and hemispheres [14,15]. In humans, controversy exists regarding when the posterior fossa should be considered large enough to qualify as DWM rather than vermian hypoplasia [14], and some authors have recommended abandonment of the term “DW variant” [10–12,15,41,42]. Furthermore, genetic studies have demonstrated that the loss of a specific gene or interstitial deletion of a chromosome can lead to variable phenotypes, ranging from mild cerebellar vermis hypoplasia to classic DWM [43,44], and their classification within the group of mesenchymal-neuroepithelial signaling defects is suggested [16,17]. Concurrent supratentorial anomalies were identified in a proportion of our dogs. Among these, hydrocephalus, which was severe in some cases, was the most common finding, followed by a subjectively thinned corpus callosum or an incomplete septum pellucidum. Enlarged lateral ventricles attributed to persistence of the fetal ventricular system rather than true hydrocephalus were reported in human reelin mutations [40], but neither hydrocephalus nor agenesis of the corpus callosum have been described in humans with VLDLR-associated cerebellar hypoplasia, while ventriculomegaly and agenesis of the corpus callosum are the most commonly identified supratentorial malformations in humans with DWM [15,42], Other malformations, such as inter-hemispheric cysts or encephaloceles, gray matter heterotopias, malformations of the dentate nucleus and brainstem, hamartomas, and lissencephaly are also described with DWM [10,15,37,38,45,46]. In the past, hydrocephalus has frequently been recognized in dogs in association with agenesis of the cerebellar vermis and a Dandy-Walker malformation was frequently suggested [18–20,22,23,24].

The data presented in our dog families suggested autosomal recessive inheritance and excluded X-linked or dominant inheritance because both sexes were affected, and both parents of one affected dog were healthy as assessed by MRI. The percentage of affected littermates was most compatible with autosomal recessive inheritance and with the observed high frequency of the VLDLR gene mutation in the population [27]. Up to now, seven different VLDLR mutations are recognized in humans. These are phenotypically and neuroanatomically indistinguishable and inherited in an autosomal recessive fashion population [33,35]. Mutations in WDR81 and the CA8 cause a similar phenotype in humans [35,47]. In contrast, the genetic basis of DWM in humans has not been completely clarified. DWM can occur as an isolated syndrome or together with clinically recognizable genetic syndromes (Meckel-Gruber and Walker-Warburg Syndrome); alternatively, DWM can have a multifactorial origin [15,48–50]. Familial cases of DWM have been described and several candidate genes for DWM have been recently uncovered. These include the cerebellar genes ZIC1 and ZIC4, FOXC1, a de novo 2.3-Mb deletion of chromosome 8p21.2-p21.3 associated with down regulation of the FGF17 gene, and ZIC2 and ZIC5 on the long arm of chromosome 13 [51–57]. In addition to these findings, DWM has been reported in a wide variety of other chromosomal anomalies [15,49,57–59]. The etiological heterogeneity and yet undefined genetic basis of DWM impair current prenatal and genetic counselling [60,61].

The outcomes of the dogs with cerebellar hypoplasia resembling DWLM varied in the present investigation. Some dogs were euthanatized as puppies presumably due to severe ataxia, but in those that continued to live with their carers the ataxia was non-progressive and did not interfere with the dogs` normal activity and behavior. Many of these affected dogs appeared to develop normally and showed a learning capacity and behavior that were normal for the breed. One dog was euthanized because of epileptic seizures that were refractory to standard antiepileptic therapy. The functional outcome of children with DWM is still poorly defined because of disease heterogeneity [15,37]. DWM usually presents as an isolated case of hydrocephalus in pediatric patients but occasionally presents clinically in adults [14,56]. Mental retardation is common in cases of a severely abnormal lobulated vermis [37]. The introduction of shunts as a surgical treatment to reestablish the posterior fossa architecture has dramatically improved the prognosis of DWM [10,14,15,56].

In summary, an inferior cerebellar hypoplasia resembling DWLM was diagnosed in purebred Eurasier dogs with early-onset, non-progressive ataxia. Pedigree data suggested autosomal recessive inheritance. Most recently, we identified a deletion in the VLDLR receptor gene based on the information provided by the dogs from the present study [27]. Eurasier dogs with cerebellar hypoplasia resembling DWLM provide a spontaneous animal model that may help to investigate the long-term consequences and cogniti-ve outcomes of this disease and provide insights into the mechanisms of cerebellar development.

## Supporting Information

S1 Fig MRI of dog 4. Midsagittal, dorsal and transverse T2W MR brain images of the caudal fossa. Corresponding levels are outlined by green lines.
(TIF)

S2 Fig MRI of dog 6. Midsagittal, dorsal and transverse T2W MR brain images of the caudal fossa. Corresponding levels are outlined by green lines.
(TIF)

S3 Fig MRI of dog 7. Midsagittal, dorsal and transverse T2W MR brain images of the caudal fossa. Corresponding levels are outlined by green lines.
(TIF)

S4 Fig MRI of dog 8. Midsagittal T2W MR brain image (littermate of dog 7; dorsal and transverse MR views are unavailable from this dog).
(TIF)

S5 Fig MRI of dog 9. Midsagittal, dorsal and transverse T2W MR brain images of the caudal fossa. Corresponding levels are outlined by green lines.
(TIF)

S6 Fig MRI of dog 10. Midsagittal, dorsal and transverse T2W MR brain images at the level of the caudal fossa. Corresponding levels are outlined by green lines.
(TIF)

S1 Table Morphometric measurements. (DOCX)

S1 Video Cerebellar ataxia in the puppies (dogs 12–14). (MP4)

S2 Video Dog 6—Subtle dysmetria and truncal sway. Dog 6 displayed only a subtle dysmetric gait, which was barely detectable. The neurologic examination revealed an absent menace response and a subtle delay in medial eye movements on examination of oculocephalic movements. This dog was followed for two years. Repeat examinations showed a still absent menace response.
(MP4)

S3 Video Dogs 7 and 8—Cerebellar ataxia in the puppies. Dogs 7 and 8 showed cerebellar ataxia of the trunk and limbs with hypermetria as puppies on initial examination.
(MP4)

S4 Video Dogs 7 and 8—Improved ataxia in the adult dogs. Reevaluation of dogs 7 and 8 at one year of age: The ataxia was barely visible when the dogs were running or walking. The dogs still had problems calculating distance and easily lost balance when jumping. Some difficulty ascending and descending stairs was observed (shown at the end of the video sequence). The menace reaction was still absent, and an intermittent spontaneous horizontal nystagmus was recorded during the neurologic examination.
(MP4)

S5 Video Dog 10—Subtle incoordination while navigating stairs. Dog 10 was presented because of a recent onset of epileptic seizures at four years of age. The owner complained about non-progressive pelvic limb ataxia since the dog was a puppy. The dog exhibited subtle thoracic limb incoordination when walking downstairs and “bunny-hopping” of the pelvic limbs when walking upstairs.
(MP4)

S6 Video Dog 11—Mild ataxia and head tilt. Dog 11 showed a mild dysmetric gait with leaning to the left and a head tilt to the left. Positional nystagmus was evident in this dog during the neurologic examination.
(MP4)

S7 Video Dog 10—Postural reactions. Dog 10 exhibited delayed initiation of movements (hemiwalk and wheelbarrowing). The pelvic limbs appeared more impaired.
(MP4)

S8 Video Dog 10—Nystagmus. Dog 10 displayed episodes with alternating directions of nystagmus and horizontal jerk nystagmus.
(MP4)
