# Supplementary figures and images for: Inferior Cerebellar Hypoplasia Resembling a Dandy-Walker-Like Malformation in Purebred Eurasier Dogs with Familial Non-Progressive Ataxia: A Retrospective and Prospective Clinical Cohort Study
Source: PLoS One. 2015 Feb 10;10(2):e0117670. doi: 10.1371/journal.pone.0117670 (PMC4323131; doi:10.1371/journal.pone.0117670)

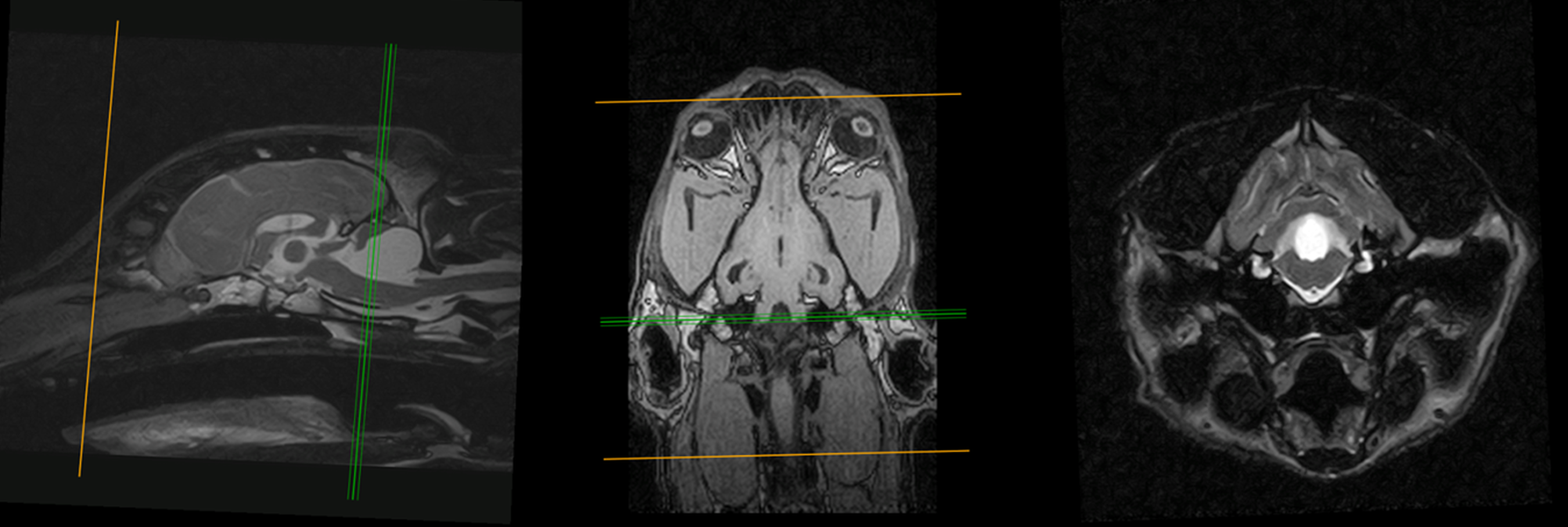

Supplement: S1 Fig — Midsagittal, dorsal and transverse T2W MR brain images of the caudal fossa. Corresponding levels are outlined by green lines. (TIF) [file pone.0117670.s001.tif]

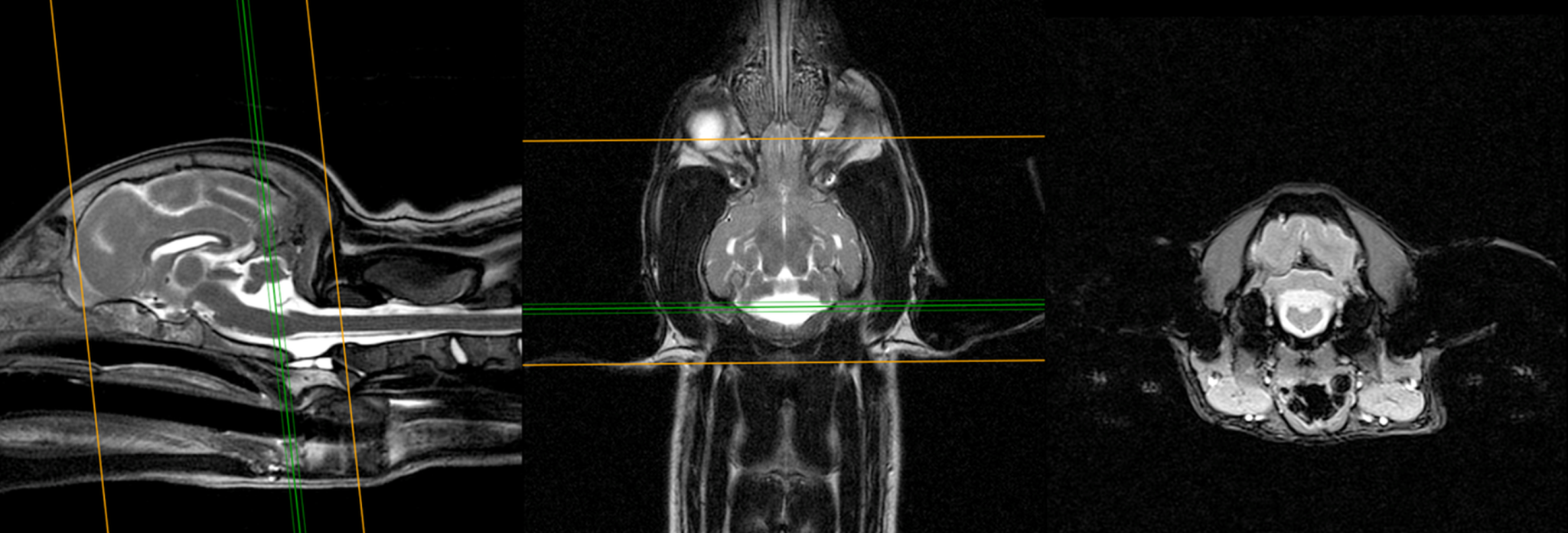

Supplement: S2 Fig — Midsagittal, dorsal and transverse T2W MR brain images of the caudal fossa. Corresponding levels are outlined by green lines. (TIF) [file pone.0117670.s002.tif]

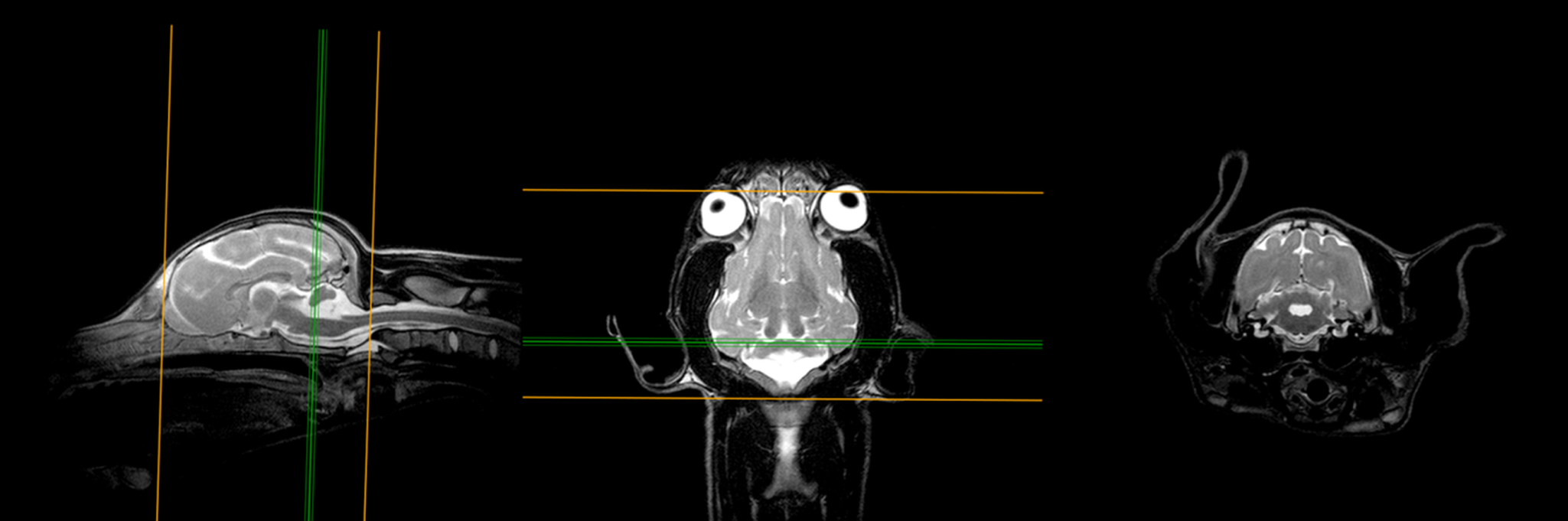

Supplement: S3 Fig — Midsagittal, dorsal and transverse T2W MR brain images of the caudal fossa. Corresponding levels are outlined by green lines. (TIF) [file pone.0117670.s003.tif]

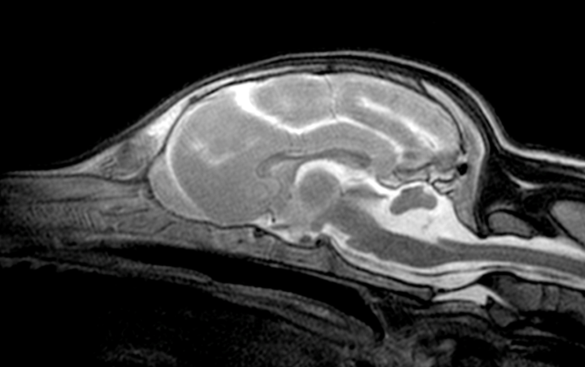

Supplement: S4 Fig — Midsagittal T2W MR brain image (littermate of dog 7; dorsal and transverse MR views are unavailable from this dog). (TIF) [file pone.0117670.s004.tif]

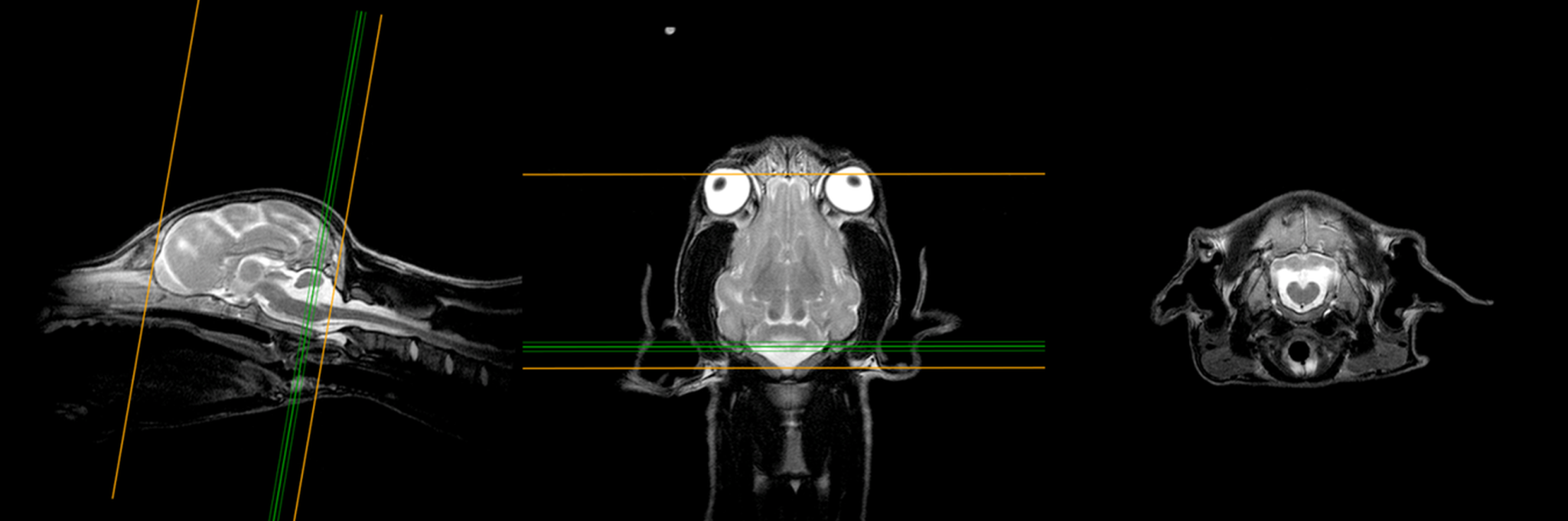

Supplement: S5 Fig — Midsagittal, dorsal and transverse T2W MR brain images of the caudal fossa. Corresponding levels are outlined by green lines. (TIF) [file pone.0117670.s005.tif]

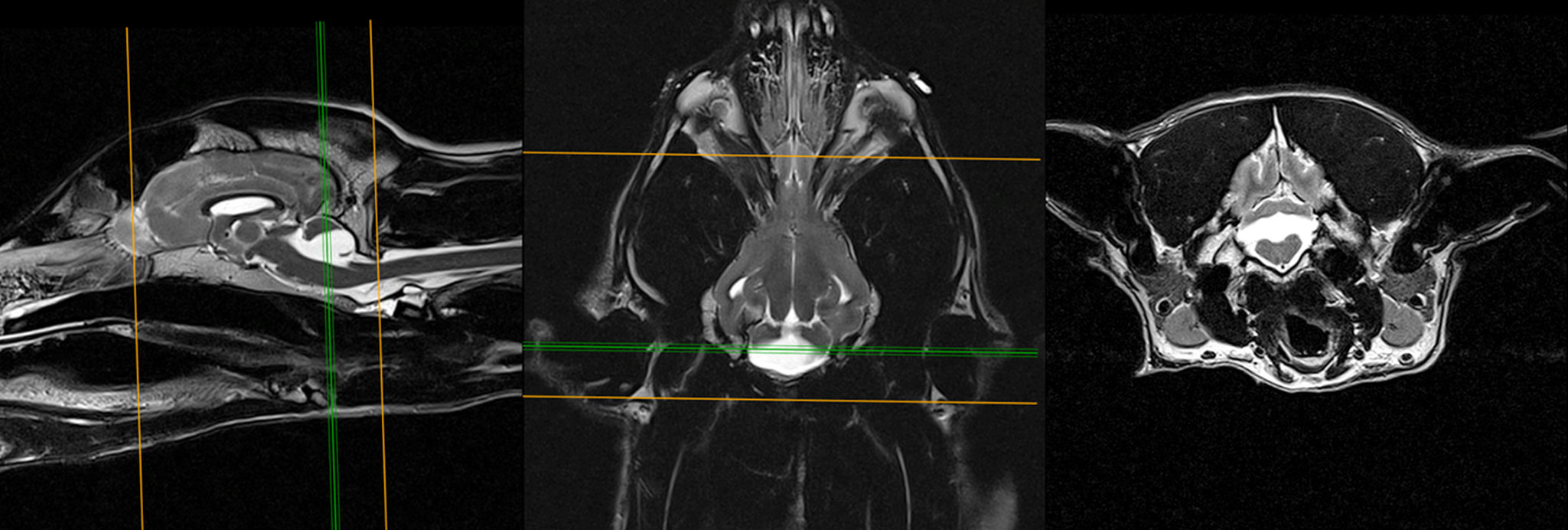

Supplement: S6 Fig — Midsagittal, dorsal and transverse T2W MR brain images at the level of the caudal fossa. Corresponding levels are outlined by green lines. (TIF) [file pone.0117670.s006.tif]
