# Supplementary material for: Inferior Cerebellar Hypoplasia Resembling a Dandy-Walker-Like Malformation in Purebred Eurasier Dogs with Familial Non-Progressive Ataxia: A Retrospective and Prospective Clinical Cohort Study
Source: PLoS One. 2015 Feb 10;10(2):e0117670. doi: 10.1371/journal.pone.0117670 (PMC4323131; doi:10.1371/journal.pone.0117670)
Supplement: S1 Table — (DOCX) [file pone.0117670.s007.docx]

| **Dog**  **No** | **Study cohort** | **Patient classification** | **Litter no** | **Phenotype** | **Caudal fossa midsagittal area**  **(cm^2^)**  **(1^st^ measure)** | **Caudal fossa midsagittal area**  **(cm^2^)**  **(2^st^ measure)** | **Total braincase midsagittal area**  **(cm^2^)**  **(1^st^ measure)** | **Total braincase midsagittal area**  **(cm^2^)**  **(2^st^ measure)** | **Caudal fossa area**  **: total braincase area**  **(1^st^ measure)** | **Caudal fossa area**  **: total braincase area**  **(2^nd^ measure)** | **Ratio**  **caudal fossa area : total braincase area**  **(mean)** |
| --- | --- | --- | --- | --- | --- | --- | --- | --- | --- | --- | --- |
| 1 | retrospective | case | 1 | ataxia | 4.063 | 4.905 | 23.191 | 23.868 | 0.175 | 0.206 | 0.1905 |
| 2 | retrospective | case | 2 | ataxia | - | - | - | - | - | - | - |
| 3* | retrospective | case | 2 | ataxia | 13.170 | 13.445 | 30.147 | 30.243 | 0.437 | 0.445 | **0.4410** |
| 4 | retrospective | case | 3 | ataxia | 8.633 | 8.723 | 29.397 | 30.171 | 0.294 | 0.289 | 0.2915 |
| 5* | retrospective | case | 3 | ataxia | 9.756* | 9.813 | 28.126 | 28.383 | 0.348 | 0.339 | **0.3435** |
| 6 | prospective | case | 4 | ataxia | 5.823 | 5.932 | 23.412 | 23.808 | 0.249 | 0.249 | 0.2490 |
| 7 | prospective | case | 5 | ataxia | 5.068 | 5.200 | 22.181 | 22.164 | 0.228 | 0.235 | 0.2315 |
| 8 | prospective | case | 5 | ataxia | 4.665 | 4.653 | 22.206 | 22.251 | 0.210 | 0.209 | 0.2095 |
| 9 | prospective | case | 6 | ataxia | 4.160 | 4.507 | 20.795 | 20.812 | 0.200 | 0.217 | 0.2085 |
| 10 | prospective | case | 3 | ataxia | 7.494 | 7.689 | 29.284 | 29.581 | 0.256 | 0.260 | 0.2580 |
| 11* | prospective. | case | 7 | ataxia | 9.652 | 9.749 | 27.482 | 27.407 | 0.351 | 0.356 | **0.3535** |
|  | prospective | littermate | 4 | healthy | 7.316 | 7.597 | 28.360 | 28.072 | 0.258 | 0.271 | 0.2645 |
|  | prospective | littermate | 4 | healthy | 10.304 | 10.473 | 32.396 | 32.457 | 0.318 | 0.323 | 0.3205 |
|  | prospective | littermate | 4 | healthy | 10.144 | 10.073 | 30.886 | 30.318 | 0.328 | 0.332 | 0.3300 |
|  | prospective | dam | 4 | healthy | 10.186 | 10.168 | 31.622 | 31.947 | 0.322 | 0.318 | 0.3200 |
|  | prospective | sire | 4 | healthy | 8.693 | 8.815 | 28.535 | 28.998 | 0.305 | 0.304 | 0.3045 |
| * | prospective | dam | 5 | healthy | 7.425 | 7.143 | 26.443 | 26.354 | 0.281 | 0.271 | 0.2760 |
|  | prospective | dam | 6 | healthy | 8.973 | 9.000 | 28.000 | 28.368 | 0.320 | 0.317 | 0.3185 |
|  | prospective | id. epilepsy |  | seizures, normal MR | 10.147 | 10.253 | 34.754 | 34.830 | 0.292 | 0.294 | 0.2930 |
|  | prospective | id. epilepsy |  | seizures. normal MR | 10.345 | 10.535 | 32.831 | 32.989 | 0.315 | 0.319 | 0.3170 |

*measurements on computed tomographic images; id. epilepsy: idiopathic epilepsy; MR: magnetic resonance brain images
